# Braided composite stent for peripheral vascular applications

**DOI:** 10.1515/ntrev-2020-0056

**Published:** 2020-11-27

**Authors:** Qingli Zheng, Pengfei Dong, Zhiqiang Li, Ying Lv, Meiwen An, Linxia Gu

**Affiliations:** Institute of Biomedical Engineering, College of Biomedical Engineering, Taiyuan University of Technology, Taiyuan, 030024, China; Department of Biomedical and Chemical Engineering and Sciences, Florida Institute of Technology, Melbourne, FL, 32901, United States of America; Institute of Applied Mechanics, College of Mechanical and Vehicle Engineering Taiyuan University of Technology, Taiyuan, 030024, China; Institute of Biomedical Engineering, College of Biomedical Engineering, Taiyuan University of Technology, Taiyuan, 030024, China; Institute of Biomedical Engineering, College of Biomedical Engineering, Taiyuan University of Technology, Taiyuan, 030024, China; Department of Biomedical and Chemical Engineering and Sciences, Florida Institute of Technology, Melbourne, FL, 32901, United States of America

**Keywords:** atherosclerosis, braided composite stent, surface coverage, radial strength, flexibility, finite element method

## Abstract

Braided composite stent (BCS), woven with nitinol wires and polyethylene terephthalate (PET) strips, provides a hybrid design of stent. The mechanical performance of this novel stent has not been fully investigated yet. In this work, the influence of five main design factors (number of nitinol wires, braiding angle, diameter of nitinol wire, thickness and stiffness of the PET strip) on the surface coverage, radial strength, and flexibility of the BCS were systematically studied using computational models. The orthogonal experimental design was adopted to quantitatively analyze the sensitivity of multiple factors using the minimal number of study cases. Results have shown that the nitinol wire diameter and the braiding angle are two most important factors determining the mechanical performance of the BCS. A larger nitinol wire diameter led to a larger radial strength and less flexibility of the BCS. A larger braiding angle could provide a larger radial strength and better flexibility. In addition, the impact of the braiding angle decreased when the stent underwent a large deformation. At the same time, the impact of the PET strips increased due to the interaction with nitinol wires. Moreover, the number of PET strips played an important role in the surface coverage. This study could help understand the mechanical performance of BCS stent and provides guidance on the optimal design of the stent targeting less complications.

## 1 Introduction

Peripheral artery disease (PAD), mostly caused by atherosclerosis, reduces the blood flow into the arteries of arms, legs, and feet, further inducing tissue damage and gangrene [1,2]. The stent implantation, due to its minimally invasive nature, is widely adopted for the treatment of PAD [3,4]. The stent is able to push the narrowed arterial wall outward and achieve immediate lumen gain [5]. However, the stent induced abnormal force on the arterial wall, which could overstimulate the proliferation of smooth muscle cells, leading to in-stent restenosis [4,6].

Much efforts have been made to optimize the stent design [7,8] since stent structure and materials influence its clinical efficacy [9–12]. The ideal stent requires adequate radial stiffness to scaffold the vessel wall [13,14], longitudinal flexibility to pass through tortuous blood vessels, and radial compliance to be conformed to the lesions [15]. Among various stents, braided stents showed superiority in terms of longitudinal flexibility and radial compliance [16–19]. Covered stents have demonstrated their efficacy in mitigating the tissue ingrowth [20,21]. But it is in the cost of a large profile and reduced flexibility [22–24].

Recently, a novel braided composite stent (BCS) was proposed to combine the merits of both braided stent and covered stent for achieving the desirable radial strength, flexibility, and increased surface coverage [25]. It was made of nitinol wires and polyethylene terephthalate (PET) helical strips. Both of these materials have been widely used in the vascular surgery products with good biocompatibility [26–28]. Braiding technique contributes to the flexibility of the BCS. Motivated by the performance of a covered stent, the PET strips, with large surface coverage, were adopted to mitigate the tissue ingrowth. The BCS exhibits more flexibility than the covered stent. Specifically, it was reported that the bending moment of the BCS was 17.4% smaller than that of the covered stent when subjected to a bending angle of 30° [25,29]. The presence of wide PET strips of BCS can constrain the relative displacement of the nitinol wires and enhance its radial strength at a large deformation [25,30]. The parameters such as nitinol wire diameter, initial braiding angle, and strip thickness are essential to the mechanical performance of the BCS. But the systematic study of the influence of BCS design parameters on its mechanical performances does not exist. In this study, an orthogonal experimental design was utilized to quantify the influence of five input factors (number of nitinol wires, braiding angle, diameter of nitinol wire, thickness and stiffness of the PET strip) that govern the material property and geometry of the BCS on the surface coverage, radial strength, and flexibility. The response to the stent implantation is patient-specific. One specific goal of this study is to identify patterns that might account for the variability of various patients. In addition, quantitative evaluation of the design sensitivity of the BCS could facilitate an optimal design of the stent.

## 2 Materials and methods

### 2.1 The geometrical design of the stent

**Figure 1:**
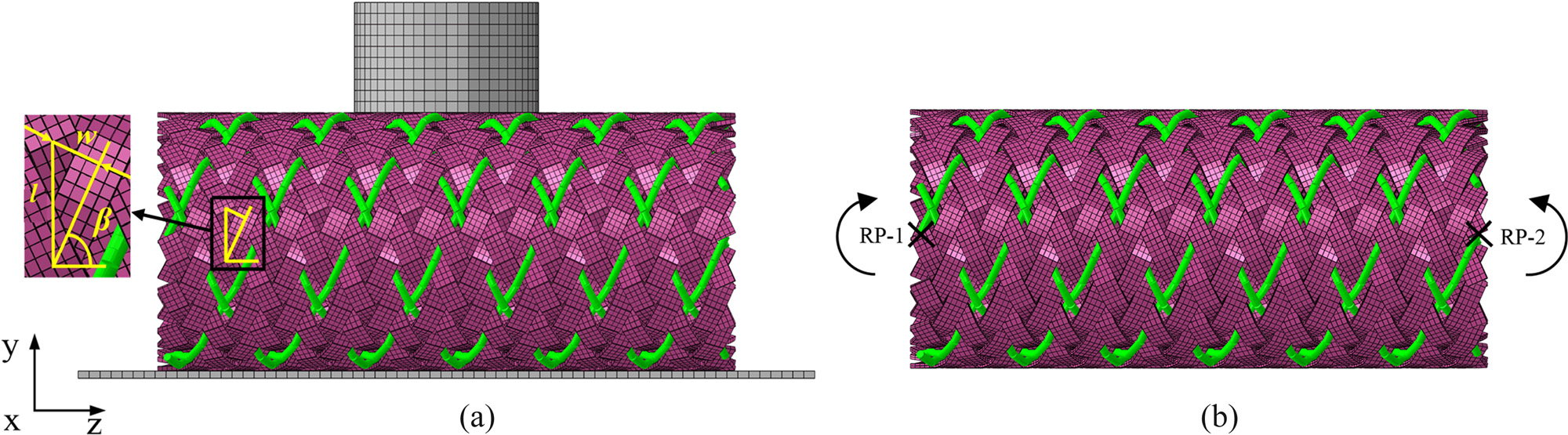
Three-dimensional model of BCS: (a) compression test and (b) bending test.

The BCS, braided with 32 strands of nitinol wires and PET strips, has a length of 15 mm and an outer diameter of 7 mm (Figure 1). The stent geometry is mainly defined by four independent parameters: number of the nitinol wires, braiding angle, diameter of the nitinol wire, and the thickness of the PET strip. Two levels of each geometrical parameter are considered, as listed in Table 1. Figure 1 shows one geometrical configuration of the stent with 8 0.15 mm-diameter nitinol wires, 24 0.1 mm-thick polyester fibers, and a braiding angle of 65°.

The braiding angle *β* is the angle between the helical wire and the axial direction of the stent. The width of the PET strip *w* could be calculated by:

(1) $$w=l\cos\beta =\frac{\pi D}{16}\cos\beta ,$$

where *l* is the distance of the two intersection points along the circumferential direction (Figure 1a) and *D* is the outer diameter of the stent. The width of PET strip is 0.775 and 0.57 mm for the braiding angle of 55° and 65°, respectively.

The surface coverage *μ* of the stent characterizes the stent porosity, which is associated with the tissue ingrowth. It is calculated by:

(2) $$\mu =\frac{L_{\text{wire}}d\;+\;L_{\text{strip}}w\;-\;NA}{\pi DL}\;\;=\frac{n_{1}d\;+\;n_{2}w}{\pi D\cos\beta }\;-\;\frac{N_{1}dw\;+\;N_{2}d^{2}\;+\;N_{3}w^{2}}{2\pi^{2}D^{2}\cos^{2}\beta }$$

where *N* refers to the number of intersections, *A* is the area of intersections; NA can be expressed as (*N*_1_*dw* + *N*_2_*d*^2^ + *N*_3_*w*^2^)/sin^2^ *β* with *N*_1_ as the number of intersections between polyester strip and nitinol wire, *N*_2_ as the number of intersections between nitinol wires, and *N*_3_ as the number of intersections between polyester strips. *L* is the length of stent, which can be expressed as *L* = π*D* cot *β*; *d* is the diameter of the nitinol wire; *w* is the width of the polyester strip; *L*_wire_ and *L*_strip_ are the total length of the nitinol wire and polyester strip, respectively. Both can be calculated based on the number of nitinol wire *n*_1_ or polyester strips *n*_2_, and the braiding angle *β* [31] as

(3) $$L_{\text{wire}}=\frac{n_{1}\pi D}{\sin\beta }\;L_{\text{strip}}=\frac{n_{2}\pi D}{\sin\beta }.$$

### 2.2 Finite element modeling

Three-dimensional models of the BCSs were constructed on the aforementioned geometrical design. The nitinol wire was adopted as a superelastic material, which was implemented through a built-in Abaqus User Material Subroutine (VUMAT) [32]. The PET strip was an elastoplastic material with Young’s modulus ranging from 2.5 to 3.5 Gpa [33], which is considered as a main factor in the study. The material parameters are summarized in Table 2.

Compression tests were carried out to investigate the influence of stent design parameters on the radial strength of the stent (Figure 1a). The stent was placed on a rigid plate (20 mm × 10 mm), which was completely fixed in space by six degrees of freedom. Specifically, the middle section of the stent was radially compressed by a rigid presser foot of 5 mm in diameter. The displacement enforced on the presser foot was 3.5 mm, namely, 50% of the outer diameter of the stent [34] and then released the presser foot at the same rate of 1.75 mm/s. The reaction force of the presser foot, i.e., load applied on the stent was monitored during the loading and unloading process.

The stent flexibility was characterized by the pure bending tests [35] (Figure 1b). A fixed planar end cross-section was enforced by means of a “rigid body” connection bound to a 6 DOF reference point at each end of the stent (RP1 and RP2). The following boundary conditions were applied, restraining five of the six degrees of freedom: (1) restrained translation in *X* and *Y* directions, (2) restrained rotation about *Y* and *Z* axes, (3) *X* deflection of RP1 + *X* deflection of RP2 = 0. (4) Rotation about *X* axes of RP1 and RP2 reached at 60° [36], respectively, in opposite directions. The bending moments applied at both ends were recorded to evaluate the flexibility of the stent.

The stent wires were meshed with two-node linear beam element (B31) and the PET strips were meshed with reduced 4-node doubly curved shell elements (S4R). The nitinol wire was meshed with 192 elements and 240 elements for the braiding angle of 55° and 65°, respectively. The PET strip was meshed with 2,176 elements and 2,160 elements for the braiding angle of 55° and 65°, respectively. The rigid compressor foot and supporting plate were meshed with 1,645 and 4,622 rigid quadrilateral R3D4 elements, respectively. Mesh-sensitivity analyses were conducted to insure that all results are convergent. A general contact algorithm was adopted among all contact surfaces with a friction coefficient of 0.3 [37]. The kinetic energy of each stent accounted for less than 5% of the internal energy to avoid the inertia effect. Finite element models were solved using a commercial Abaqus/Explicit software (Dassault Systèmes Simulia Corp.).

### 2.3 Design of computational experiments

An orthogonal experimental design Ln(*t^q^*) was applied to identify the sensitivity of multiple factors with minimal experiments, where *L* is the orthogonal array, *n* is the number of study cases, *t* is the level of factors, and *q* is the number of factors [38]. In this work, a five-factor two-level array L_8_(2^5^) was designed to systematically evaluate the sensitivities of five input factors to the mechanical performance of the stent. The modulus of the PET strip and four geometrical design parameters (number of nitinol wire *n*, initial braiding angle *β*, nitinol wire diameter *d*, and PET strip thickness *t*) levels of each input factor are shown in Table 1. The study cases designed according to the L_8_(2^5^) orthogonal table are shown in Table 3. The surface coverage, compression load, and bending moment of the study cases were examined.

## 3 Results

The validation of the BCS models had been conducted in our previous work [25]. The radial strength of the stent was evaluated while compressing the stent to 50% of the original diameter of 7 mm, and the numerical result agreed well with the experimental result [25,30].

The surface coverage, the compression load, and the bending moment for all eight cases are summarized in Table 4. The range analysis was conducted to quantify the influence of each factor on the mechanical performance of the stent, as shown in Tables 5 and 6. *K*_1_ and *K*_2_ represent the influence of the lower level and higher level of a specific factor on the result measurement, respectively. A specific factor *K_i_* value is the average of four values of the result (Table 4) with level *i* (Table 3). The range value *R* of each factor is the difference between the *K*_1_ and *K*_2_. The larger *R* value demonstrates that the corresponding factor is more sensitive to the mechanical performance of the stent. The influence of each factor in the calculation results was finally normalized, according to the ratio between the values of the high and low levels.

**Figure 2:**
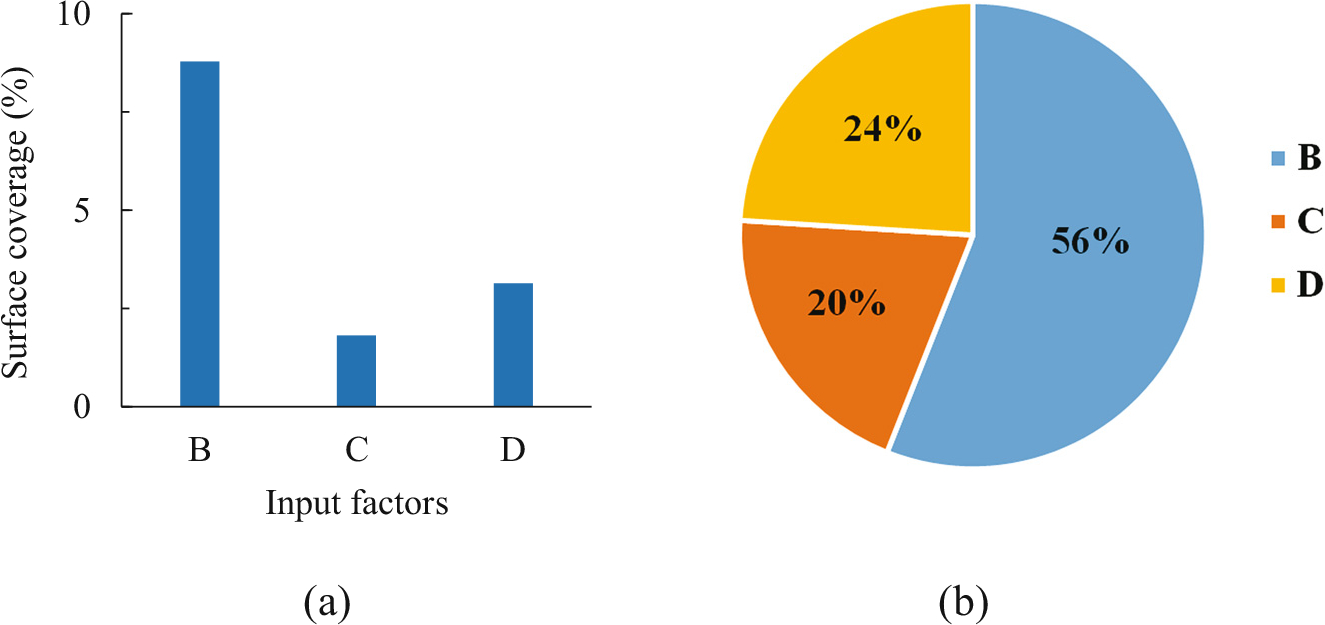
Diagram of the range value on surface coverage (a) and normalized (b) at the initial state to various input factors.

The surface coverage, an important parameter of stent for preventing the growth tissue from entering the stent, is presented in Table 4. As expected, more PET strips give higher surface coverage. Therefore, it is mainly related to the number of nitinol wire (B, 56%) of the stent (Figure 2). If the PET strips in case 2 (97.3%) and case 6 (96.8%) are changed into nitinol wires with a diameter of 0.15 mm, the surface coverage of stent is greatly reduced, which is only 46.7% and 35.8%, respectively. In addition, the effect of initial braiding angle and diameter of the wire on surface coverage of the stent is relatively small (20% vs 24%). The surface coverage is an important character to be considered when comparing a composite stent with a bare metal stent.

**Figure 3:**
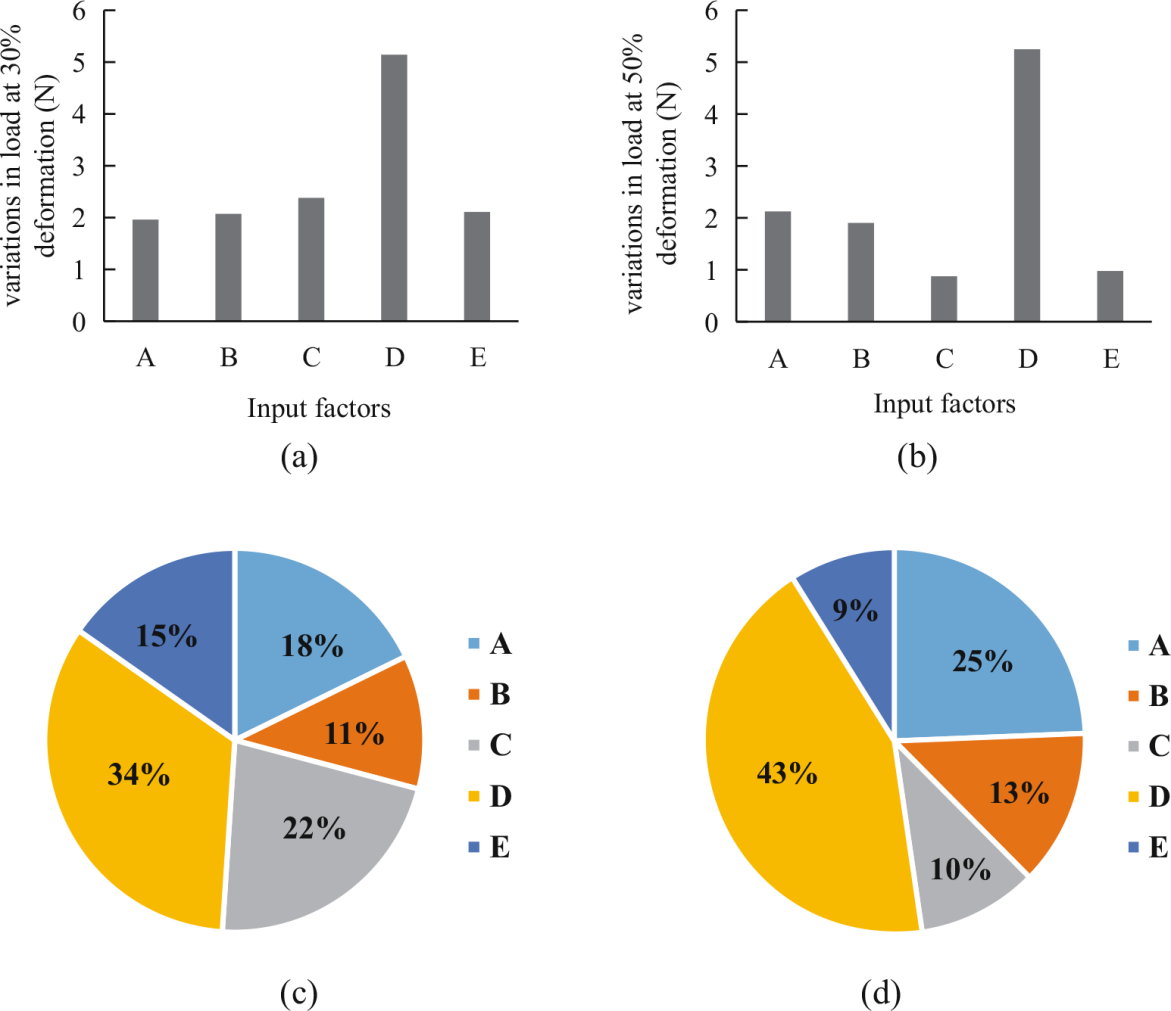
Diagram of the range value on compression load at 30% (a) and 50% (b) deformation of the original diameter and normalized at 30% (c) and 50% (d) of diameter deformation to various input factors. A– PET strip modulus (GPa), B– number of nitinol wire, C– initial braiding angle (°), D– nitinol wire diameter (mm), and E– PET strip thickness (mm).

The compression load at the deformation of 30% and 50% of its original diameter during the loading process for all eight cases is summarized in Table 4. The load increases with the increase of compression displacement. It is clear that factor *D* (nitinol diameter) was the most sensitive one to the compression load among the five factors during the loading process no matter at the small or large deformation (Figure 3), and the influence is getting more important as the deformation increased from 30% to 50%, i.e., the range value normalized to radial strength increased by 9% (34–43%). While the importance of factor *C* (initial braiding angle) reduced from 22% to 10% at the large deformation, and the value of the factor A (PET strip modulus) becomes prominent from 18% to 25%. The reason is that the radial strength is mainly borne by nitinol wires during the whole compression process, while with the increase of compression deformation, the braiding angle under the presser foot becomes smaller, the PET strips enforced more constrain on the nitinol wires and enhanced the radial strength of the stent.

**Figure 4:**
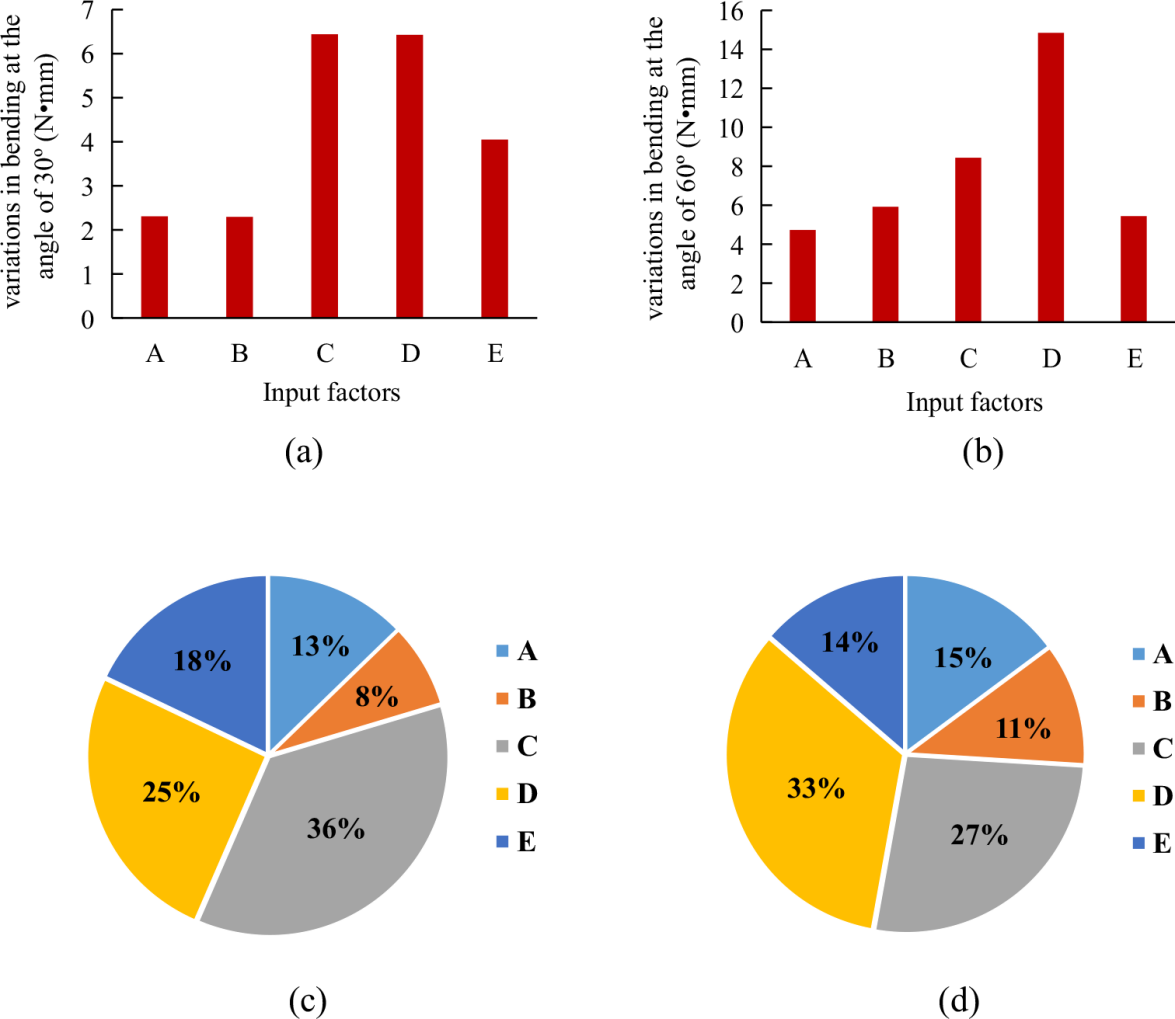
Diagram of the range value on the bending moment at the bending angle of 30° and 60° and normalized at 30° (c) and 60° (d) to various input factors. A– PET strip modulus (GPa), B– number of nitinol wire, C– initial braiding angle (°), D– nitinol wire diameter (mm), and E– PET strip thickness (mm).

**Figure 5:**
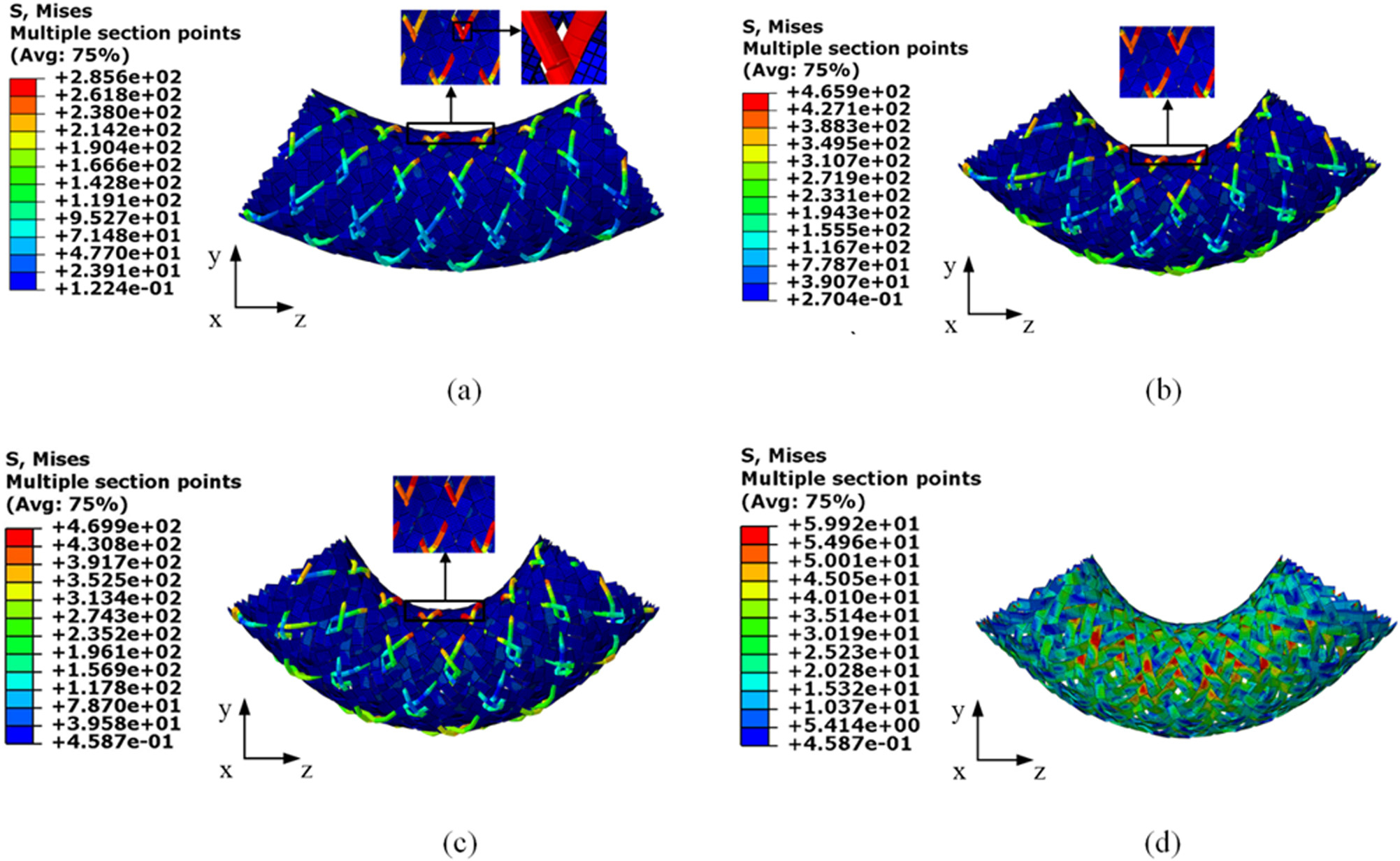
Stent deformation of case 1 at the bending angle of 30° (a), 48° (b), 60° (c) (the zoom-in view on the *XZ* planes), and (d) PET strips at the bending angle of 60°.

The bending moments at the bending angles of 30° and 60° for all eight cases are summarized in Table 4. The greater bending moment indicated a worse flexibility of the stent. The sensitivities of the five input factors to bending moment are depicted in Figure 4. The proposed results clearly highlight that the bending moment depends strongly on the factor *C* (initial braiding angle) and factor *D* (nitinol diameter). Bigger initial braiding angle can achieve better flexibility of the stent, while the increase of the nitinol diameter can reduce the flexibility of the stent. In detail, the initial braiding angle is more sensitive to the bending moment at the bending angle of 30° (36%), while this sensitivity decreases (27%) when the bending angle is up to 60°. By contrast, the influence of the nitinol wire diameter on the bending moment becomes more prominent with the bending angle increase from 30° (25%) to 60° (33%) (Figure 4). It can be explained by the deformed configurations of the stent, in case 1 (Figure 5). It is clear that nitinol wires and PET strips have relative sliding during the bending process. The stress concentrations occur in the region where the stent is under bending and compression. From the *XZ* plane shown in Figure 5, it can be seen that the porosity of the stent in the bending and compression areas becomes smaller when the bending angle increases, and it reaches a critical value at the bending angle of 48°, and then, the sliding between nitinol wires and PET strips weakens and the importance of the initial braiding angle to the bending moment decreases. It is clear that the whole stent material undergoes an elastic state during the bending process. The peak von Mises stress of nitinol wires is 469.9 MPa at the plateau of phase transformation between austenite and martensite of nitinol and that of PET strips is 59.92 MPa, which well below the ultimate material strength (Figure 5c and d). The bending moment is mainly borne by nitinol wires.

**Figure 6:**
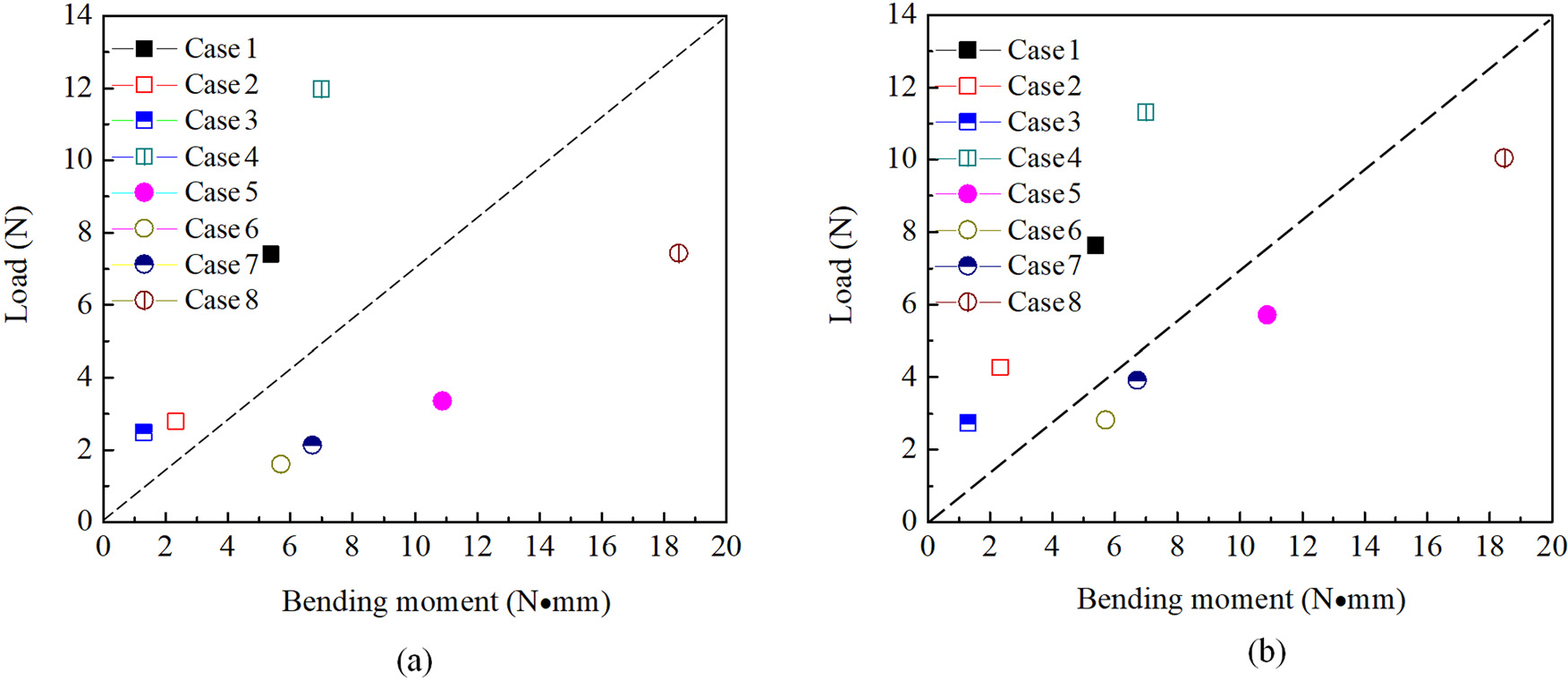
Combination diagrams of the bending moment at bending angle of 30° and compression load at the 30% (a) and 50% (b) deformation of the original diameter.

To comprehensively evaluate the mechanical properties of the stents in this article, the bending moment as the abscissa axis and radial strength as the ordinate axis were combined, as shown in Figure 6. The model on the upper left of the dotted line shown in the figure is the braiding angle of 65°, while the lower right is the braiding angle of 55°. This indicate that the mechanical performances of the stents with a large braiding angle are better than those with a small braiding angle. The larger radial strength corresponds to the model with larger nitinol wire diameter in the diagram (case 1, 4, 5, and 8.).

## 4 Discussion

For peripheral artery stenosis, the implanted stent will be exposed to various “dynamic” loads, such as radial compression, bending, and torsion [27,39]. The dynamic physiological environment requires both the radial strength and the flexibility of the stent, which motivated the design of a novel hybrid BCS. In this work, mechanical behaviors of the BCS were systematically investigated through computational models. An orthogonal experimental design was adopted to quantify the influence of five input factors on the surface coverage, the radial strength, and the flexibility of the stent.

The radial strength is the primary consideration of the stent conceptual design to scaffold the narrow lumen [8]. The nitinol diameter is the most critical factor among the five factors for the radial strength of the BCS. A larger nitinol diameter and the initial braiding angle resulted in a higher radial strength, which applies to all braided stent including the BCS in this article and braided nitinol stent (BNS) [37]. While in BNS, the nitinol diameter has less influence on the radial strength than the initial braiding angle [35]. The influence of factor difference between two stents can be explained by the unique structure of BCS. In BCS, the presence of wide PET strips can constrain the sliding of the nitinol wires and enhance the radial strength of the stent at the large deformation, the radial strength was 52% larger than that of the BNS at the deformation of 50% original diameter [25]. Due to the interaction between the nitinol wire and PET strips, the structure of BCS tends to be more stable. Meanwhile, due to its wide PET strips, the BCS can provide a higher surface coverage that could be considered as a potential alternative to the covered stent, which has a larger radial strength while compromising its flexible performance [24]. The mechanical properties of PET strip could be enhanced by using a drug-loading nanocomposite, which will be investigated in the future work [40].

The longitudinal flexibility of stent was associated with the risk of kinking and incidence of limb thrombosis in tortuous anatomy [15]. Without enough flexibility, the stent does not make full contact with the artery wall when there is knee flexion and this may increase the risk of restenosis due to the interaction between the stent and the artery [41,42]. For BCS, the bigger initial braiding angle can result in a better flexibility, whereas the larger nitinol wire diameter can induce a worse flexibility of stent. The most critical factor for the bending moment of the BCS is the initial braiding and then changed to the nitinol wire diameter when the bending angle changes from 30° to 60°, which is different from the BNS that the nitinol diameter is most sensitive to the bending moment during the whole bending process [35]. The difference may result from the presence of PET strips, which could reduce the sliding between nitinol wires at a small bending angle. The porosity becomes smaller with an increase of the bending angle in the bending and compression areas until they squeeze each other (Figure 5). After that the bending moment is mainly borne by nitinol wires. BCS exhibits better flexibility than the BNS and the covered stent at a small bending angle, i.e., the bending angle at 30° [25,29].

## 5 Conclusions

The BCS has advantages of good flexibility and high radial strength. To optimize the stent design in different physiological environments is urgently needed based on the understanding the sensitivity of stent parameters. In this study, the orthogonal experimental design was adopted to quantify the influence of five input factors on the surface coverage, radial strength, and flexibility of the stent. Our results show that nitinol diameter and initial braiding angle are two most sensitive factors that affect the mechanical performance of BCS. The influence of the nitinol diameter increased when the stent underwent a large deformation, while the influence of the initial braiding angle was the opposite. At the same time, the impact of PET strips increased due to the interaction with nitinol wires. The surface coverage of BCS mainly depends on the number of the PET strips. In addition, the interaction between stent and a torturous artery in a complex mechanical environment should also be carried out simultaneously to better evaluate the mechanical properties of the stent.

## Figures and Tables

**Table 1:** Orthogonal experimental factors and levels

Type	Symbol	Input factors	Levels	
			Low	High

Material property	A	PET strip modulus *E* (GPa)	2.5	3.0
Geometry	B	Number of nitinol wire *n*	8	16
	C	Initial braiding angle *β* (°)	55	65
	D	Nitinol wire diameter *d* (mm)	0.15	0.25
	E	PET strip thickness *t* (mm)	0.1	0.15

**Table 2:** Material properties of nitinol and PET

Nitinol	
Austenite elasticity *E*_A_ (MPa)	50,000
Martensite elasticity *E*_M_ (MPa)	37,000
Start of transformation loading $\sigma_{\text{U}}^{\text{S}}$ (MPa)	400
End of transformation loading $\sigma_{\text{L}}^{\text{E}}$ (MPa)	650
Start of transformation unloading $\sigma_{\text{U}}^{\text{S}}$ (MPa)	350
End of transformation unloading $\sigma_{\text{U}}^{\text{E}}$ (MPa)	80
Volumetric transformation strain $\varepsilon_{\text{V}}^{\text{L}}$	0.055
PET	
Young’s modulus *E* (GPa)	2.5/3.0
Poisson ratio *v*	0.4
Yield stress *σ*_s_ (MPa)	60
Tensile strength *σ*_t_ (MPa)	70

**Table 3:** Scheme of orthogonal experimental design

Case no.	Input factors				
	A	B	C	D	E

1	2.5	8	65	0.25	0.15
2	3	8	65	0.15	0.15
3	2.5	16	65	0.15	0.1
4	3	16	65	0.25	0.1
5	2.5	8	55	0.25	0.1
6	3	8	55	0.15	0.1
7	2.5	16	55	0.15	0.15
8	3	16	55	0.25	0.15

**Table 4:** Surface coverage, compression load, and bending moment of the cases

Case no.	Surface coverage (%)	Load (N)		Bending moment (N mm)	
		30%	50%	30°	60°

1	98.6	7.391	7.625	5.396	15.070
2	97.3	2.780	4.268	2.338	7.356
3	87.6	2.472	2.731	1.302	4.344
4	93.1	11.963	11.301	7.013	18.364
5	97.8	3.349	5.705	10.892	20.125
6	96.8	1.596	2.814	5.713	7.610
7	85.0	2.127	3.907	6.722	13.004
8	89.7	7.427	10.040	18.481	38.137

**Table 5:** Range analysis for the compression load at 30% and 50% compression deformation

Input factors	Load at 30% deformation (N)			Load at 50% deformation (N)		
	*K*_1_	*K*_2_	*R*	*K*_1_	*K*_2_	*R*

A	3.835	5.796	1.961	4.992	7.116	2.124
B	3.779	5.851	2.072	5.103	7.005	1.902
C	6.006	3.625	2.381	6.492	5.617	0.875
D	2.244	7.387	5.143	3.430	8.678	5.248
E	5.733	3.625	2.109	6.593	5.617	0.977

**Table 6:** Range analysis for the bending moment at the bending angle of 30° and 60°

Input factors	Bending moment at the angle of 30° (N mm)			Bending moment at the angle of 60° (N mm)		
	*K*_1_	*K*_2_	*R*	*K*_1_	*K*_2_	*R*

A	6.078	8.386	2.308	13.136	17.867	4.731
B	6.085	8.379	2.295	12.540	18.462	5.922
C	4.012	10.452	6.440	11.283	19.719	8.436
D	4.019	10.445	6.427	8.078	22.924	14.846
E	6.402	10.452	4.050	14.278	19.719	5.441
